# The Relationship Between Inter-limb Asymmetry and Sprinting Performance in Sprint Athletes: A Systematic Review

**DOI:** 10.1186/s40798-026-01082-1

**Published:** 2026-10-01

**Authors:** Joachim D’Hondt, Laurent Chapelle, Jarno Van Handenhoven, Jasper Stalmans, Dirk Aerenhouts

**Affiliations:** Movement and Nutrition for Health and Performance (MOVE) Research Group, Department of Movement and Sport Sciences, Faculty of Physical Education and Physiotherapy, Vrije Universiteit Brussel, Pleinlaan 2, 1050 Brussels, Belgium

**Keywords:** Side-to-side differences, Bilateral difference, Between-limb, Functional asymmetry, Morphological asymmetry, Kinematic asymmetry, Kinetic asymmetry, Biomechanics, Sprinting

## Abstract

**Background:**

Inter‑limb asymmetries are commonly observed in athletes, yet their relationship with sprinting performance remains poorly understood. While most existing research has focused on unilateral sports, a comprehensive systematic review examining the relationship between lower limb asymmetry and sprinting performance in sprint athletes is still lacking.

**Objective:**

This systematic review aimed to examine the association between lower inter-limb asymmetry and sprinting performance in sprint athletes.

**Methods:**

A systematic search was conducted in PubMed, Scopus, SPORTDiscus and Web of Science to identify studies investigating lower inter-limb asymmetry and sprinting performance in healthy, injury-free sprint athletes. The review was conducted in accordance with PRISMA 2020 guidelines (PROSPERO: CRD420251140520). Study quality was assessed using the Downs and Black Quality Index Tool, whilst the evidence certainty was evaluated with the GRADE framework.

**Results:**

Of 4,811 articles screened, 10 studies met the inclusion criteria. In general, limited associations were observed between functional, morphological, kinetic and kinematic asymmetry and sprinting performance. However, task- and metric-specific associations were observed in individual studies.

**Conclusions:**

Given the limited number of eligible studies and the substantial heterogeneity in study designs, tasks, and asymmetry metrics, drawing definitive conclusions remains challenging. The evidence synthesized in this review is mixed, and therefore does not allow for a straightforward assertion that inter-limb asymmetry is associated with reduced sprinting performance. To enhance comparability and clarify the potential role of inter-limb asymmetry in sprinting, future research should implement standardized, task-specific, metric-specific, and muscle-specific assessment approaches.

**Supplementary Information:**

The online version contains supplementary material available at https://doi.org/10.1186/s40798-026-01082-1.

## Key Points

- The inconsistent evidence on the relationship between inter‑limb asymmetry and sprinting performance in sprint athletes indicates that reducing asymmetry does not necessarily need to be a central focus of training.
- Future longitudinal research is needed to investigate a possible cause and effect relationship between inter-limb asymmetry and sprinting performance using task-, metric-, segment-, individual-, and time-specific approaches.
- Employing standardized asymmetry calculations is essential for establishing meaningful inter-limb asymmetry threshold ranges.

**Supplementary Information:**

The online version contains supplementary material available at https://doi.org/10.1186/s40798-026-01082-1.

## Background

Symmetry refers to the equal size, shape, and positioning of corresponding body structures relative to a central axis [1]. In the context of human movement, deviations from symmetry between limbs, referred to as inter-limb asymmetry, are common and may be associated with both structural biomechanical alignment and functional performance [2]. Inter-limb asymmetry is typically categorized into different types including functional (e.g., strength or jump performance), morphological (e.g., differences in limb length or lean mass), kinematic (e.g., discrepancies in joint angles, step length, or stride time) and kinetic asymmetries (e.g., imbalances in ground reaction forces or impulse production) [2–6].

Sprinting performance is defined as the ability to achieve and maintain maximal running velocity over short distances (typically up to 400 m) [7]. Muscle strength and power are two important determinants of sprinting performance which are strongly influenced by structural characteristics, such as muscle size, muscle quality, fascicle length, and pennation angle [8–10]. Sprinting relies on the coordinated contribution of force and power from each leg. Research has shown that the combined bilateral output during sprinting and jumping tasks is not simply equal to the sum of each leg’s individual capacity [11, 12]. At the same time, sprinting kinematics often exhibit inter-limb asymmetries [13], raising the question of whether such asymmetries might, under certain conditions, impair performance.

Existing research on the relationship between inter-limb asymmetry and sprinting performance has primarily focused on team-sports, where unilateral performance determinants are prominent [5, 14–17]. For instance, Bishop et al. [14] demonstrated a strong positive association between single-leg countermovement jump (SLCMJ) asymmetry and 5 m (*r* = 0.86, *p* < 0.001), 10 m (*r* = 0.87, *p* < 0.001) and 20 m (*r* = 0.79, *p* < 0.001) sprinting times in U16 soccer players from an English Premier League soccer academy. Similarly, a study on 81 young elite team-sport athletes of different sport disciplines found a significant positive association between single‑leg countermovement jump (SLCMJ) asymmetry and 30 m sprinting time (*r* = 0.26, *p* < 0.05) [16]. A recent systematic review with meta-analysis by Fox et al. [18] also reported a small but significant negative association between lower-limb asymmetry and sprinting performance (*r* = 0.20), although most eligible studies included unilateral sports and were of low methodological quality.

Collectively, these findings suggest that inter-limb asymmetry may be negatively associated with sprinting performance. However, it is important to recognize that much of the existing research has been conducted in athletes practising unilateral (team) sports, who are inherently more prone to developing greater asymmetries due to the repetitive, sport-specific unilateral loading patterns involved [3, 19]. However, previous research has also shown that asymmetries can develop in bilateral sports such as endurance running and sprinting, arising from the preferential use of one side of the body during repetitive cyclic movement patterns [20–24]. A recent systematic review by D’Hondt et al. [23] reported that inter-limb asymmetries are generally either negatively or not significantly associated with performance-related outcomes in endurance runners. These findings indicate that inter-limb asymmetry may also be negatively associated with performance in cyclical sports such as running. However, due to the biomechanical and neuromuscular differences between endurance running and sprinting [23], it remains unclear whether inter-limb asymmetry is associated with sprinting performance in sprint athletes.

To address these gaps, a sprinting-specific synthesis of the evidence is warranted to determine whether and how inter-limb asymmetry is associated with sprinting performance. Accordingly, the present systematic review aimed to comprehensively evaluate and appraise the available literature examining inter-limb asymmetry and its associations with sprinting performance and its determinants in sprint athletes.

## Methods

This systematic review followed the Preferred Reporting Items for Systematic Reviews and Meta-Analyses (PRISMA 2020) [25], and was registered online on PROSPERO on 4 September 2025 (ID: CRD420251140520).

### Eligibility Criteria

In this systematic review, all articles published up to 17 March 2026 were eligible for inclusion. Only original peer‑reviewed studies were considered. A summary of the eligibility criteria applied during the systematic search, structured according to the PECOS (population, exposure, comparators, outcomes, study design) framework [25], is provided in Table 1. No specific comparison (C) was applied.

**Table 1:** Eligibility criteria

	Inclusion criteria
Population	• Healthy human male and female sprint athletes of any age and level, and free from injury (in cases where studies included both injured and injury-free individuals, the results for the injury-free participants were analysed)
Exposure	• Tests designed to assess inter‑limb asymmetries
Outcome of interest	• Analysis of the association between lower inter‑limb asymmetry and both linear and curvilinear sprinting performance (and/or its determinants), including sprint distances up to 400 m
Study design	• Peer-reviewed articles including interventional and observational study designs

### Information Sources and Search Strategy

The literature search was conducted across 4 four electronic databases, including PubMed, Scopus, SPORTDiscus and Web of Science on 17 March 2026. In all data bases, articles were searched using title and abstract fields. The search strategy was developed using predefined keywords and MeSH terms based on the PECOS framework (Table 1). No limit was imposed on publication date. Finally, backward citation tracking and forward citation tracking using Web of Science was performed. The full search strings as well as the retrievals for each data base are presented in Table 2.

**Table 2:** Search strategy for each database with the corresponding number of retrieved records

Database	Retrievals	Full search strategy using Boolean operators across all databases (*N* = 4)
PubMed	876	(sprint* Or "track and field" OR “track & field” OR athletics) AND ("functional laterality" OR balance OR imbalance OR asymmetr* OR symmetr* OR dissymmetr* OR "bilateral difference" OR interlimb OR "inter-limb" OR inequalit* OR "between-limb" OR "between limb") AND (performance OR speed OR sprint* OR acceleration OR velocity OR "stride length" OR "step length" OR "contact time" OR "cost of running" OR "energy cost")
Scopus	1,611	
SPORTDiscus	1,521	
Web of Science	2,796	

### Study Selection Process

The study selection and data collection process were performed blindly by 2 independent researchers (J.D. and J.V.H.). The articles were assembled from the four databases. Subsequently, all duplicates were removed prior to screening using the EndNote software. Additionally, all articles were first screened on title and abstract and then on full text using the Rayyan software [26].

### Data Collection Process

Data were extracted by two authors (J.D and J.V.H.), who performed this process independently. A standardized data extraction form was developed in Microsoft Word to systematically record information from each included study. Extracted details encompassed publication year, study design, and participant characteristics (including sample size, mean age, sex distribution, and training status according to McKay’s framework [27]). Additionally, information was collected on the metrics and formulas used to quantify inter-limb asymmetry, and any reported relationships between inter-limb asymmetry and measures of running performance. For intervention studies, associations from all reported time points were considered.

### Methodological Quality

Methodological quality was independently evaluated by two researchers (J.D. and J.V.H). Any discrepancies were resolved through consensus or, when necessary, by consultation with a third researcher (D.A.). To ensure the quality of the included studies, a systematic assessment of the methodological quality was conducted using the Downs and Black Quality Index Tool [28]. Following the approach of two recent systematic reviews [23, 29], a modified version of the tool was employed, including only items relevant to the scope of this review. Specifically, questions related to patient treatment, training interventions, and group randomization were omitted. Each of the remaining 10 items (see Table 3) was scored as 1 (yes = ‘•’), 0 (no = ‘○’), or ‘-’ when the study report did not provide sufficient information to assign a score. Following the rationale of previous work, the overall methodological quality scores were interpreted using three categories: (1) low quality for scores of 5/10 or below; (2) good quality for scores of 6/10 to 7/10; and (3) excellent quality for scores of 8/10 or higher [30].

**Table 3:** Modified Downs and Black checklist [1] items used for methodological quality assessment of included studies

Item number	Question
*Reporting*	
**1**	**Is the hypothesis/aim/objective of the study clear?**
**2**	**Are the main outcomes to be measured clearly described in the introduction or methods section?** *Information outlined in introduction/methodology for both physical characteristics and sprinting performance measure used for associative analysis pertaining to assessment(s) used, any calculations used and units of measurement
**3**	**Are the characteristics of the subjects included in the study clearly described?** *Source defined, with characteristics included
**4**	**Are the main findings of the study clearly described?**
**5**	**Does the study provide estimates of the random variability in the data for the main outcomes?** *One of the following included for both physical characteristics and sprinting performance measures: a) mean ± *SD, b) standard error, c) confidence intervals and d) interquartile range*
**6**	**Have actual probability values been reported (e.g., 0.035 rather than < 0.05) for the main outcomes except where the probability value is less than 0.001?** *Exact correlation (r) and significance (p) values provided, specific to the associative analysis
*External validity*	
**7**	**Were the subjects to participate in the study representative of the entire population from which they were recruited?** * Proportion of subjects asked to participate, relative to the sample population, explicitly stated. Unless evident, then answer "unable to determine"
*Internal validity*	
**8**	**If any of the results of the study were based on 'data dredging', was this made clear** *Were any additional data analysis reported in the results not highlighted during the methodology
**9**	**Were statistical tests used to assess the main outcomes appropriate**
**10**	**Were the main outcome measures accurate (valid and reliable)?**

### Certainty of Evidence

The Grading of Recommendations, Assessment, Development, and Evaluation (GRADE) framework was applied to assess the certainty of evidence for each outcome across the included studies [31].

## Results

### Study Selection

**Fig. 1:**
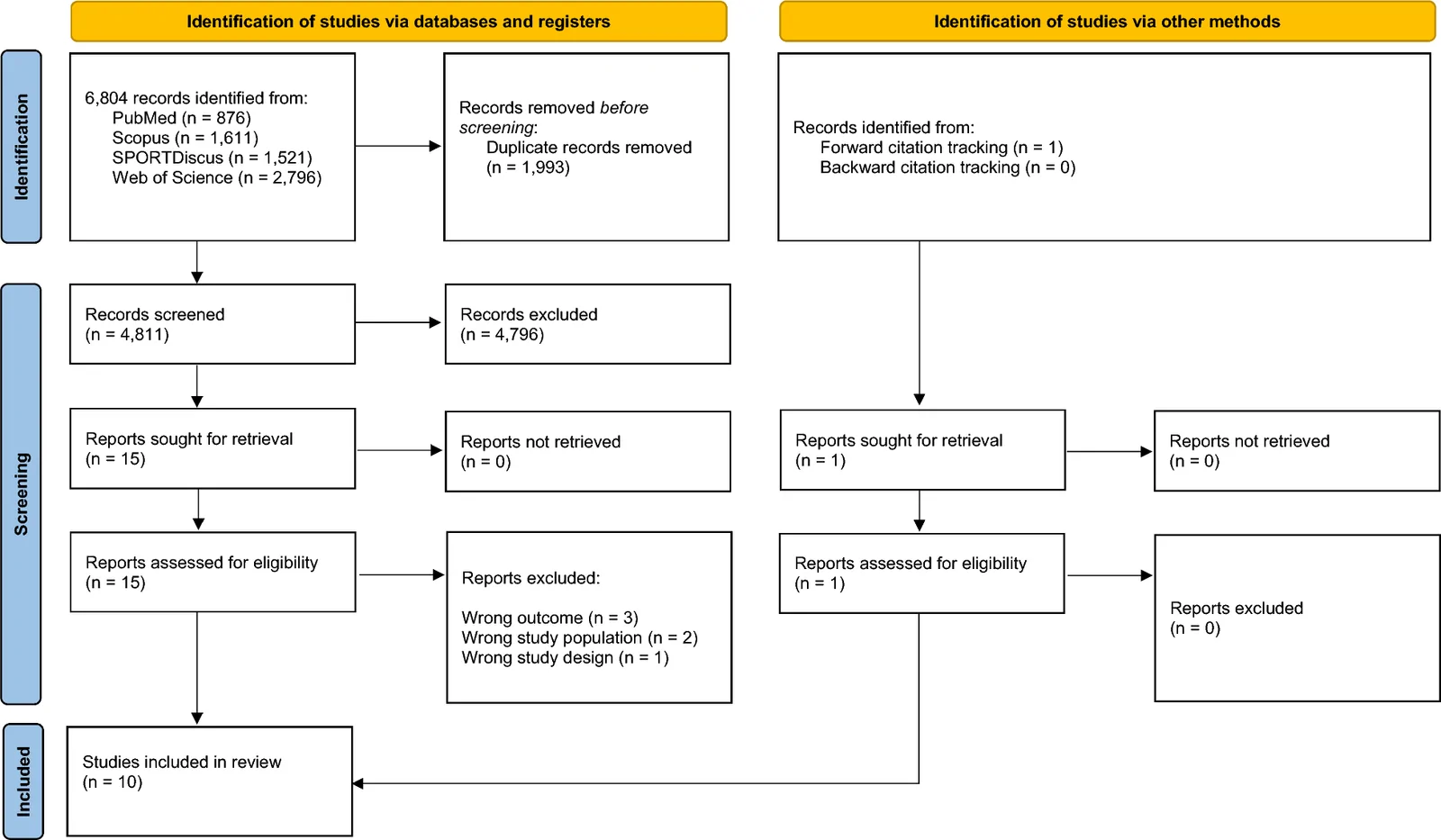
Flow diagram

In total, 6804 studies were identified through the systematic search strategy across the databases PubMed (*n* = 876), Scopus (*n* = 1,611), SPORTDiscus (*n* = 1,521) and Web of Science (*n* = 2,796). After removing duplicates, 4,811 unique articles remained. These were subjected to title and abstract screening, during which 4796 studies were excluded based on irrelevance to the predefined inclusion and exclusion criteria. This left 15 studies for full-text assessment. During the full-text review, six studies were excluded. Specifically, three studies were excluded due to reporting the wrong outcome, two studies were excluded because they included the wrong population, and one study was excluded due to an incorrect study design (see Table S1). In addition, one extra study was identified through forward and backward citation tracking. Ultimately, 10 studies met all inclusion criteria and were included in the systematic review with meta-analysis (see Fig. 1) [6, 13, 32–39].

### Methodological Quality

Table 4 presents the results of the methodological quality evaluation. Because none of the studies provided details on the proportion of participants recruited compared to the overall target population, external validity could not be established. Regarding internal validity, no substantial bias was observed, although several studies did not report whether the outcome measures had been tested for validity or reliability (criteria 10). Overall, methodological quality scores for the included studies ranged from 6/10 to 8/10.

**Table 4:** Results of the methodological quality assessment for all included studies

Study	Modified Downs and Black checklist item number										Total score out of 10
	Reporting						External validity	Internal validity			
	1	2	3	4	5	6	7	8	9	10	
Bissas et al. [13]	○	○	•	•	•	•	-	•	•	•	7
Exell et al. [38]	•	•	•	•	•	○	-	•	•	-	7
Gleadhill et al. [40]	•	•	•	•	•	•	-	•	•	-	8
Gołaś et al. [39]	•	•	•	•	•	○	-	•	•	•	8
Haugen et al. [41]	•	•	•	•	•	○	-	•	•	•	8
Nagahara et al. [6]	•	•	•	•	•	•	-	•	•	-	8
Prvulović et al. [42]	•	•	•	•	•	○	-	•	•	-	7
Tottori et al. [43]	•	•	•	•	•	•	-	○	•	•	8
Trivers et al. [44]	•	○	•	•	•	•	-	○	•	-	6
Trivers et al. [45]	○	•	•	•	○	•	-	•	•	-	6

*•* = *yes; ○* = *no;—*= *unable to determine*

### Certainty of Evidence

The certainty of evidence across all outcomes was rated as very low, largely due to methodological limitations and imprecision. As no randomized controlled trials were identified, all evidence began at a low level of certainty, consistent with GRADE guidance for observational study designs [41]. The certainty of evidence was then evaluated across five domains: risk of bias, inconsistency, indirectness, imprecision, and publication bias [42, 44]. Downgrading or maintaining certainty was determined based on the presence and severity of concerns within these domains. In line with the approach outlined in a recent systematic review [45], the overall Downs and Black score was used to evaluate the risk of bias. Studies scoring below 85% were downgraded by one level, whereas those scoring below 70% were downgraded by two levels. All outcomes were downgraded by one level for risk of bias. Indirectness was considered negligible because the included study populations, exposures, and outcomes closely matched our predefined eligibility criteria [45]. Publication bias was not assessed, as no analysis met the minimum threshold of at least 10 studies required for a meaningful evaluation [40]. Moreover, because only a single study contributed data to each finding, inconsistency could not be examined. Finally, imprecision was evaluated based on sample size and the certainty of effect estimates; analyses including fewer than 800 participants in total were downgraded by one level [43]. All outcomes were downgraded by one level for imprecision, as the total sample size for each analysis was below 800 participants.

### Study Characteristics

Among the included studies, 50.0% were conducted in Europe (United Kingdom, Poland, Norway and Serbia) [13, 32, 34–36], 20.0% in North America (Jamaica) [38, 39], 10.0% in Oceania (Australia) [33], and 20.0% in Asia (Japan) [6, 37]. All studies were published between 2013 and 2025.

### Sample Characteristics

Table 5 presents an overview of the study characteristics. Across the 10 included studies, a total of 684 participants were assessed: 324 (47.4%) were male [6, 32, 33, 37, 38], 265 (38.7%) were female [6, 13, 34, 36–38], and the sex of 95 participants (13.9%) was not reported [35]. Reported mean ages ranged from 15.9 years [36] to 26.7 years [13]. In total, the included studies comprised 16 world class sprinters (9.9%, of whom 50.0% were female) [13], 8 elite level sprinters (4.9%, of whom 100% were female) [34], and 138 highly trained sprinters (85.2%, of whom 46.4% were female) [6, 32, 33, 35–37, 39]. In one particular study, the training level was not specified [38].

**Table 5:** GRADE assessment of certainty of evidence

Correlation variables	*k(n)*	Risk of bias	Indirectness	Risk of publication bias	Inconsistency	Imprecision	Certainty of evidence
Sprint time—100 m sprint							
Foot vertical velocity pre-TD asymmetry	1*(16)*	− 1 level	− 0 level	NA	NA	− 1 level	⊕
Shank angle TD asymmetry	1*(16)*	− 1 level	− 0 level	NA	NA	− 1 level	⊕
Sprint time—50 m sprint							
EMG quadriceps asymmetry	1*(8)*	− 1 level	− 0 level	NA	NA	− 1 level	⊕
EMG hamstrings asymmetry	1*(8)*	− 1 level	− 0 level	NA	NA	− 1 level	⊕
EMG gluteus asymmetry	1*(8)*	− 1 level	− 0 level	NA	NA	− 1 level	⊕
Sprint time—cross-directional difference							
Psoas major cross-sectional area L3–L4	1*(13)*	− 1 level	− 0 level	NA	NA	− 1 level	⊕
Psoas major cross-sectional area L4–L5	1*(13)*	− 1 level	− 0 level	NA	NA	− 1 level	⊕
Psoas major cross-sectional area L5–S1	1*(13)*	− 1 level	− 0 level	NA	NA	− 1 level	⊕
Psoas major cross-sectional area SUM	1*(13)*	− 1 level	− 0 level	NA	NA	− 1 level	⊕
Quadriceps femoris cross-sectional area 30%	1*(13)*	− 1 level	− 0 level	NA	NA	− 1 level	⊕
Quadriceps femoris cross-sectional area 50%	1*(13)*	− 1 level	− 0 level	NA	NA	− 1 level	⊕
Quadriceps femoris cross-sectional area 70%	1*(13)*	− 1 level	− 0 level	NA	NA	− 1 level	⊕
Quadriceps femoris cross-sectional area SUM	1*(13)*	− 1 level	− 0 level	NA	NA	− 1 level	⊕
Hamstrings cross-sectional area 30%	1*(13)*	− 1 level	− 0 level	NA	NA	− 1 level	⊕
Hamstrings cross-sectional area 50%	1*(13)*	− 1 level	− 0 level	NA	NA	− 1 level	⊕
Hamstrings cross-sectional area 70%	1*(13)*	− 1 level	− 0 level	NA	NA	− 1 level	⊕
Hamstrings cross-sectional area SUM	1*(13)*	− 1 level	− 0 level	NA	NA	− 1 level	⊕
Sprint time—average between counter-clockwise and clockwise directions							
Psoas major symmetry	1*(13)*	− 1 level	− 0 level	NA	NA	− 1 level	⊕
Quadriceps femoris symmetry	1*(13)*	− 1 level	− 0 level	NA	NA	− 1 level	⊕
Hamstrings symmetry	1*(13)*	− 1 level	− 0 level	NA	NA	− 1 level	⊕
Mean velocity—60 m sprint							
Net ankle work asymmetry	1*(8)*	− 1 level	− 0 level	NA	NA	− 1 level	⊕
Maximum vertical force asymmetry	1*(8)*	− 1 level	− 0 level	NA	NA	− 1 level	⊕
Kinematic asymmetry	1*(8)*	− 1 level	− 0 level	NA	NA	− 1 level	⊕
Kinetic asymmetry	1*(8)*	− 1 level	− 0 level	NA	NA	− 1 level	⊕
Kinematic asymmetry composite score	1*(8)*	− 1 level	− 0 level	NA	NA	− 1 level	⊕
Kinetic asymmetry composite score	1*(8)*	− 1 level	− 0 level	NA	NA	− 1 level	⊕
Mean velocity—90 m sprint							
Composite score asymmetry	1*(288)*	− 2 levels	− 0 level	NA	NA	− 1 level	⊕
Knee joint width asymmetry	1*(288)*	− 2 levels	− 0 level	NA	NA	− 1 level	⊕
Ankle joint width asymmetry	1*(288)*	− 2 levels	− 0 level	NA	NA	− 1 level	⊕
Foot length asymmetry	1*(288)*	− 2 levels	− 0 level	NA	NA	− 1 level	⊕
Mean velocity—100 m sprint							
Explosive strength asymmetry	1(18)	− 1 level	− 0 level	NA	NA	− 1 level	⊕
Mean velocity—180 m sprint							
Composite score asymmetry	1*(288)*	− 1 level	− 0 level	NA	NA	− 1 level	⊕
Knee joint width asymmetry	1*(288)*	− 1 level	− 0 level	NA	NA	− 1 level	⊕
Ankle joint width asymmetry	1*(288)*	− 1 level	− 0 level	NA	NA	− 1 level	⊕
Foot length asymmetry	1*(288)*	− 1 level	− 0 level	NA	NA	− 1 level	⊕
Changes in mean velocity—60 m sprint							
Running velocity asymmetry	1*(13)*	− 1 level	− 0 level	NA	NA	− 1 level	⊕
Step length asymmetry	1*(13)*	− 1 level	− 0 level	NA	NA	− 1 level	⊕
Step frequency asymmetry	1*(13)*	− 1 level	− 0 level	NA	NA	− 1 level	⊕
Stance time asymmetry	1*(13)*	− 1 level	− 0 level	NA	NA	− 1 level	⊕
Flight time asymmetry	1*(13)*	− 1 level	− 0 level	NA	NA	− 1 level	⊕
Braking impulse asymmetry	1*(13)*	− 1 level	− 0 level	NA	NA	− 1 level	⊕
Propulsive impulse asymmetry	1*(13)*	− 1 level	− 0 level	NA	NA	− 1 level	⊕
Anteroposterior net impulse asymmetry	1*(13)*	− 1 level	− 0 level	NA	NA	− 1 level	⊕
Vertical impulse asymmetry	1*(13)*	− 1 level	− 0 level	NA	NA	− 1 level	⊕
Braking mean force asymmetry	1*(13)*	− 1 level	− 0 level	NA	NA	− 1 level	⊕
Propulsive mean force asymmetry	1*(13)*	− 1 level	− 0 level	NA	NA	− 1 level	⊕
Anteroposterior net mean force asymmetry	1*(13)*	− 1 level	− 0 level	NA	NA	− 1 level	⊕
Vertical mean force asymmetry	1*(13)*	− 1 level	− 0 level	NA	NA	− 1 level	⊕
Braking time asymmetry	1*(13)*	− 1 level	− 0 level	NA	NA	− 1 level	⊕
Propulsive time asymmetry	1*(13)*	− 1 level	− 0 level	NA	NA	− 1 level	⊕
Countermovement jump height asymmetry	1*(13)*	− 1 level	− 0 level	NA	NA	− 1 level	⊕
Maximal velocity—60 m sprint							
Running velocity asymmetry	1*(64)*	− 1 level	− 0 level	NA	NA	− 1 level	⊕
Step length asymmetry	1*(64)*	− 1 level	− 0 level	NA	NA	− 1 level	⊕
Step frequency asymmetry	1*(64)*	− 1 level	− 0 level	NA	NA	− 1 level	⊕
Step width asymmetry	1*(64)*	− 1 level	− 0 level	NA	NA	− 1 level	⊕
Stance time asymmetry	1*(64)*	− 1 level	− 0 level	NA	NA	− 1 level	⊕
Flight time asymmetry	1*(64)*	− 1 level	− 0 level	NA	NA	− 1 level	⊕
Braking pulse asymmetry	1*(64)*	− 1 level	− 0 level	NA	NA	− 1 level	⊕
Propulsive impulse asymmetry	1*(64)*	− 1 level	− 0 level	NA	NA	− 1 level	⊕
Medial impulse asymmetry	1*(64)*	− 1 level	− 0 level	NA	NA	− 1 level	⊕
Lateral impulse asymmetry	1*(64)*	− 1 level	− 0 level	NA	NA	− 1 level	⊕
Mediolateral net impulse asymmetry	1*(64)*	− 1 level	− 0 level	NA	NA	− 1 level	⊕
Anteroposterior net impulse asymmetry	1*(64)*	− 1 level	− 0 level	NA	NA	− 1 level	⊕
Vertical impulse asymmetry	1*(64)*	− 1 level	− 0 level	NA	NA	− 1 level	⊕
Braking mean force asymmetry	1*(64)*	− 1 level	− 0 level	NA	NA	− 1 level	⊕
Propulsive mean force asymmetry	1*(64)*	− 1 level	− 0 level	NA	NA	− 1 level	⊕
Medial mean force asymmetry	1*(64)*	− 1 level	− 0 level	NA	NA	− 1 level	⊕
Lateral mean force asymmetry	1*(64)*	− 1 level	− 0 level	NA	NA	− 1 level	⊕
Mediolateral net mean force asymmetry	1*(64)*	− 1 level	− 0 level	NA	NA	− 1 level	⊕
Anteroposterior net mean force asymmetry	1*(64)*	− 1 level	− 0 level	NA	NA	− 1 level	⊕
Vertical mean force asymmetry	1*(64)*	− 1 level	− 0 level	NA	NA	− 1 level	⊕
Braking time asymmetry	1*(64)*	− 1 level	− 0 level	NA	NA	− 1 level	⊕
Propulsive time asymmetry	1*(64)*	− 1 level	− 0 level	NA	NA	− 1 level	⊕
				NA	NA	− 1 level	⊕
Lactate 1 min post-set				NA	NA	− 1 level	⊕
EMG quadriceps asymmetry	1*(8)*	− 1 level	− 0 level	NA	NA	− 1 level	⊕
EMG hamstrings asymmetry	1*(8)*	− 1 level	− 0 level	NA	NA	− 1 level	⊕
EMG gluteus asymmetry	1*(8)*	− 1 level	− 0 level	NA	NA	− 1 level	⊕
Lactate 4 min post-set							
EMG quadriceps asymmetry	1*(8)*	− 1 level	− 0 level	NA	NA	− 1 level	⊕
EMG hamstrings asymmetry	1*(8)*	− 1 level	− 0 level	NA	NA	− 1 level	⊕
EMG gluteus asymmetry	1*(8)*	− 1 level	− 0 level	NA	NA	− 1 level	⊕
Step velocity—18 m sprint							
Step velocity asymmetry	1*(22)*	− 1 level	− 0 level	NA	NA	− 1 level	⊕
Step length asymmetry	1*(22)*	− 1 level	− 0 level	NA	NA	− 1 level	⊕
Step frequency asymmetry	1*(22)*	− 1 level	− 0 level	NA	NA	− 1 level	⊕
Contact time asymmetry	1*(22)*	− 1 level	− 0 level	NA	NA	− 1 level	⊕
Flight time asymmetry	1*(22)*	− 1 level	− 0 level	NA	NA	− 1 level	⊕
Touchdown angle asymmetry	1*(22)*	− 1 level	− 0 level	NA	NA	− 1 level	⊕
Inter-thigh angle at TD asymmetry	1*(22)*	− 1 level	− 0 level	NA	NA	− 1 level	⊕
TO angle asymmetry	1*(22)*	− 1 level	− 0 level	NA	NA	− 1 level	⊕
Thigh angle at TO asymmetry	1*(22)*	− 1 level	− 0 level	NA	NA	− 1 level	⊕
Knee angle at TO asymmetry	1*(22)*	− 1 level	− 0 level	NA	NA	− 1 level	⊕
Maximal thigh flexion asymmetry	1*(22)*	− 1 level	− 0 level	NA	NA	− 1 level	⊕
Range of thigh motion asymmetry	1*(22)*	− 1 level	− 0 level	NA	NA	− 1 level	⊕
Knee flexion at maximal thigh extension asymmetry	1*(22)*	− 1 level	− 0 level	NA	NA	− 1 level	⊕
Horizontal ankle velocity asymmetry	1*(22)*	− 1 level	− 0 level	NA	NA	− 1 level	⊕
IAAF scores							
Composite score asymmetry	1*(73)*	− 1 level	− 0 level	NA	NA	− 1 level	⊕
Knee joint width asymmetry	1*(73)*	− 1 level	− 0 level	NA	NA	− 1 level	⊕
Ankle joint width asymmetry	1*(73)*	− 1 level	− 0 level	NA	NA	− 1 level	⊕
Foot length asymmetry	1*(73)*	− 1 level	− 0 level	NA	NA	− 1 level	⊕

*k* = number studies, *n* = number of participants, NA = not applicable, ⊕ = very low certainty of evidence, TD = touchdown, EMG = textile electromyography, TO = toe-off

### Test and Outcome Measures

#### Asymmetry Tests and Metrics

In total, three studies examined functional asymmetry [33, 34, 36], three studies morphological asymmetry [37–39], five studies kinematic asymmetry [6, 13, 32, 33, 35] and three studies kinetic asymmetry [6, 32, 33] in relation to sprinting performance or its determinants. Functional asymmetry was evaluated using a SLCMJ, while morphological asymmetry was assessed using textile electromyography (EMG) (i.e. quadriceps femoris, hamstrings, gluteal asymmetry) [34], magnetic resonance imaging (i.e. asymmetry in the cross-sectional area of the psoas major, quadriceps femoris and hamstrings) [37], and anthropometric parameters (i.e. knee and ankle joint widths as well as foot length) [38, 39]. Kinematic outcomes (e.g., spatiotemporal parameters such as contact time and step velocity) and kinetic outcomes (e.g., net horizontal and vertical impulse) were all recorded during sprinting [6, 13, 32, 33, 35].

#### Equations for Calculating Asymmetry

Across the 10 included studies, different equations were used to quantify inter-limb asymmetry (Table 5). Three studies expressed kinematic asymmetry using the symmetry angle [13, 32, 33]. Two studies calculated asymmetry as a percentage difference based on superior and inferior values [6, 35], two studies assessed fluctuating asymmetry [38, 39], and three studies calculated percentage differences directly from right- versus left-leg values [34, 36, 37]. Additionally, four studies employed composite scores to determine the overall magnitude of inter-limb asymmetry [32, 37–39].

#### Sprinting Performance-Related Metrics

Performance outcomes in sprinting were categorized into two groups: (1) determinants of sprinting performance (e.g., maximal sprinting velocity) and (2) actual performance indicators (e.g., sprinting times). Among the determinants, maximal [6] and mean [32, 33, 36, 38] velocity on different sprinting distances (e.g., 60 m, 90 m, 10 m, 180 m) was most frequently assessed. Step velocity on an 18-m sprinting distance was also examined as a determinant of sprinting performance [35]. Furthermore, cross-directional differences in sprinting times were also determined in one study [37]. Actual performance was measured either as sprinting time in standardized tests (e.g., 50 or 100 m sprinting) [13, 34, 37] or through International Association of Athletics Federations (IAAF) scores based on personal best performances [39].

### The Association Between Inter-limb Asymmetry and Sprinting Performance-Related Metrics

#### Functional Inter-limb Asymmetry Linked to Sprinting Performance-Related Metrics

Prvulović et al. [36] reported no significant association (*p* > 0.05) between mean velocity in a 100 m sprinting and the degree of explosive strength asymmetry, as measured by a SLCMJ. Although Gleadhill et al. [33] reported a moderate positive correlation (*r* = 0.429) between variability in SLCMJ jump‑height asymmetry and corresponding variability in mean 60 m sprinting velocity, this relationship did not reach statistical significance (*p* = 0.137). In contrast, Gołaś et al. [34] reported that higher lactate accumulation during repeated sprints was associated with reduced textile EMG inter-limb asymmetry of the quadriceps and gluteus muscles (*rₛ* = − 0.43 to − 0.59, *p* = 0.0003 to 0.013). They also observed weak-to-moderate positive associations between sprinting time and textile EMG asymmetry in the quadriceps, hamstrings, and gluteus muscles (*rₛ* = 0.36 to 0.59) [34].

#### Morphological Inter-limb Asymmetry Linked to Sprinting Performance-Related Metrics

Tottori et al. [37] found no significant associations between cross‐sectional area asymmetry of the psoas major, quadriceps femoris, or hamstrings muscles and average sprinting time on a circular track. Similarly, no significant relationship was identified between the cross-sectional area of these muscles and cross-directional differences in sprinting time, with the exception of inter-limb asymmetry in the summed psoas major cross-sectional area, which was negatively associated with cross-directional sprinting time differences (*r* = − 0.203, *R*^2^ = 0.04, *p* = 0.026).

Trivers et al. [38, 39] reported significant associations between anthropometric asymmetry and sprinting performance. Knee joint width asymmetry was positively associated with mean velocity over 90 m sprints (*R*^2^ = 0.04, *β* = 0.21 to 0.24, *p* = 0.01) as observed on two different occasions (i.e. 1996 and 2006), as well as with 180 m sprints in 1996 (*R*^2^ = 0.03, *β* = 0.19, *p* = 0.03). Conversely, knee joint width asymmetry was negatively associated with IAAF scores (*R*^2^ = 0.05, *β* = − 0.22, *p* = 0.04). No significant association was found between knee joint width asymmetry and mean velocity in the 180 m sprint in 2006 (*R*^2^ = 0.01, *β* = 0.14, *p* = 0.10) [38]. Furthermore, no significant associations were observed between sprinting performance (mean velocity over 90 m and 180 m sprints or IAAF scores) and ankle joint width asymmetry (*R*^2^ = 0.03, *β* = − 0.17 to 0.07, *p* = 0.087 to 0.98) or foot length asymmetry (*R*^2^ = 0.01, *β* = − 0.11 to − 0.09, *p* = 0.18 to 0.43) [38, 39]. Finally, a composite asymmetry score based on knee joint width, ankle joint width, and foot length was significantly associated with both IAAF scores (*R*^2^ = 0.05, *p* = 0.031) and mean velocity in the 90 m (*R*^2^ = 0.02 to 0.03, *β* = 0.014 to 0.17, *p* = 0.05 to 0.09) and 180 m (*R*^2^ = 0.03, *β* = 0.17 to 0.18, *p* = 0.03 to 0.04) sprints [38, 39].

#### Kinematic Inter-limb Asymmetry Linked to Sprinting Performance-Related Metrics

Bissas et al. [13] reported that the 100 m sprinting time was significantly negatively associated with foot vertical velocity pre-touchdown asymmetry in males (*r* = − 0.86, *p* = 0.01) and positively associated with shank angle touchdown asymmetry in females (*r* = 0.81, *p* = 0.02). Regarding spatiotemporal factors, no significant associations were observed between asymmetry in step frequency (*r* = 0.09 to − 0.09, *p* > 0.05), flight time (*r* = − 0.31 to − 0.14, *p* > 0.05), or step length (*r* = − 0.10 to − 0.06, *p* > 0.05) asymmetry and step velocity on an 18-m sprint, mean velocity during a 60-m sprint maximal sprinting velocity in a 60-m sprint or 100-m sprint time [6, 13, 33, 35]. Similarly, inter-limb asymmetry in other spatiotemporal variables (e.g., step width, contact time, and stance time) and kinematic variables (e.g., knee and thigh angle at toe-off) was not significantly associated with sprinting performance (*p* > 0.05) [6, 13, 32, 33, 35]. Likewise, overall kinematic asymmetry assessed using a composite score showed no significant association with mean velocity during a 60-m sprint (*p* = 0.19) [32].

#### Kinetic Inter-limb Asymmetry Linked to Sprinting Performance-Related Metrics

Net ankle work asymmetry (*r* = 0.90) and maximum vertical force asymmetry (*r* = 0.761) were significantly positively associated with mean velocity over a 60-m sprint [32]. In contrast, Nagahara et al. [6] found no significant associations between maximal sprinting velocity over 60 m and any of the kinetic asymmetry metrics assessed (e.g., braking impulse and propulsive impulse) (*r* = − 0.15 to 0.38, *p* = 0.14 to 0.92). Likewise, a composite score for kinetic asymmetry was not significantly associated with mean velocity over a 60-m sprint (*p* = 0.40) [32]. Similarly, in Gleadhill et al. [33] demonstrated that inter-limb differences in braking, propulsive, anteroposterior, vertical impulse and mean force as well as braking time and propulsive impulse, were not significantly associated with mean differences in running velocity during a 60-m sprint (Table 6).

**Table 6:** Summary of study sample characteristics, outcome measures, asymmetry calculations, inter-limb asymmetry magnitudes, and main results of the association between inter-limb asymmetry and sprinting performance-related metrics

Study and country (ISO code)	Sample characteristics	Asymmetry metric	Sprinting performance outcome measure(s)	Inter-limb asymmetry calculations	Magnitude of inter-limb asymmetry	Associations with sprinting performance
Bissas et al. [13] GBR	*n* = 16 ♂: 8 ♀: 8 Age: ♂: 26.1 ± 4.8 ♀: 26.7 ± 2.0 Training status: world class	Asymmetry in: Contact time Step frequency, width, velocity length and time DCM TD Flight time Swing-to-swing time DCM TO Hip angle TD and TO Ankle angle TD and TO Thigh angle TD and TO Trunk angle TD and TO Knee angle TD and TO Shank angle TD and TO Thigh separation angle TD and TO CM height TD and TO CM height minimum CM horizontal velocity	100 m sprint time	${\theta}_{\mathrm{S}\mathrm{Y}\mathrm{M}} = \left(\frac{45^\circ -\mathrm{a}\mathrm{r}\mathrm{c}\mathrm{t}\mathrm{a}\mathrm{n}\left(\frac{X\mathrm{l}\mathrm{e}\mathrm{f}\mathrm{t}}{Xright}\right)}{90^\circ }\right)\times 100$	Ranging between 0.3 and 36.0%	100 m sprinting time Vs. foot vertical velocity pre-TD asymmetry in males: *r* = − 0.86; *p* = 0.01 Vs. shank angle TD asymmetry in females: *r* = 0.81, *p* = 0.02 Vs. no other associations of interest were significant in either sex (*p* > 0.05)
Exell et al. [36] GBR	*n* = 8 ♂: 8 ♀: 0 Age: ♂: 22.0 ± 5.0 ♀: NA Training status: highly trained	Kinematic asymmetry in: Step velocity Step length Step frequency Minimum hip height Maximum knee lift Maximum hip Height Minimum knee angle Maximum hip extension Touchdown distance Kinetic asymmetry in: Net horizontal impulse Net vertical impulse Maximal vertical force Mean support moment Net ankle work Net knee work Net hip work Strength asymmetry (during squat jumps) in: Peak vertical force Peak power net work	Mean velocity during a 60 m sprint	${\theta}_{\mathrm{S}\mathrm{Y}\mathrm{M}} = \left(\frac{45^\circ -\mathrm{a}\mathrm{r}\mathrm{c}\mathrm{t}\mathrm{a}\mathrm{n}\left(\frac{X\mathrm{l}\mathrm{e}\mathrm{f}\mathrm{t}}{X\mathrm{r}\mathrm{i}\mathrm{g}\mathrm{h}\mathrm{t}}\right)}{90^\circ }\right)\times 100$ $\mathrm{K}\mathrm{M}\mathrm{A}\mathrm{S}= \sum_{i=1}^{n}\left|\mathrm{K}\mathrm{M}\mathrm{A}\mathrm{S}\left({x}_{n}\right)\right|$	Kinematic asymmetry: Step velocity: 8.6% to 10.2% Step length: 0.2% to 0.8% Step frequency: 0.6% to 1.7% Minimum hip height: 0.2% to 0.7% Maximum knee lift: 0.6% to 1.8% Minimum knee angle: 1.4% to 4.1% Maximum hip extension: 0.2% to 1.2% Touchdown distance: 1.8% to 6.7% Kinetic asymmetry: Net horizontal impulse: 0.2% to 25.1% Net vertical impulse: 0.1% to 6.0% Maximal vertical force: 0.4% to 4.3% Mean support moment: 2.7% to 5.3% Net ankle work: 6.1% to 93.2% Net knee work: 8.5% to 79.6% Net hip work: 3.4% to 66.4% Strength asymmetry: Peak vertical: force: 0.2% to 1.7% Peak power: 0.2% to 3.2% Net work: 0.1% to 2.3%	Mean velocity on 60 m sprint Vs. net ankle work asymmetry: *r* = 0.90 Vs. maximum vertical force asymmetry: *r* = 0.76 Vs. kinematic asymmetry: *p* > 0.05 Vs. kinetic asymmetry: *p* > 0.05 vs. kinematic asymmetry composite score: *p* = 0.19 Vs. kinetic asymmetry composite score: *p* = 0.40
Gleadhill et al. [42] AUS	*n* = 13 ♂: 13 ♀: 0 Age: ♂: 20.3 ± 1.0 ♀: NA Training status: highly trained	Kinematic asymmetry in: Running velocity Step length Step frequency Stance time Flight time Kinetic asymmetry in: braking impulse Propulsive impulse Anteroposterior net impulse Vertical impulse Braking mean force Propulsive mean force Anteroposterior net mean force Vertical mean force Braking time Propulsive time Functional asymmetry in: Countermovement jump height	Mean running velocity during a 60 m sprint	${\theta}_{\mathrm{S}\mathrm{Y}\mathrm{M}} = \left(\frac{45^\circ -\mathrm{a}\mathrm{r}\mathrm{c}\mathrm{t}\mathrm{a}\mathrm{n}\left(\frac{X\mathrm{l}\mathrm{e}\mathrm{f}\mathrm{t}}{X\mathrm{r}\mathrm{i}\mathrm{g}\mathrm{h}\mathrm{t}}\right)}{90^\circ }\right)\times 100$	Kinematic asymmetry in: Running velocity: 0.01 ± 0.05 Step length: − 0.10 ± 1.75 Step frequency: 0.11 ± 1.80 Stance time: − 0.06 ± 1.24 Flight time: − 0.17 ± 2.74 Kinetic asymmetry in: braking impulse: − 0.72 ± 5.71 Propulsive impulse: − 0.63 ± 2.26 Anteroposterior net impulse: < 0.01 ± 18.74 Vertical impulse: − 0.80 ± 2.49 Braking mean force: − 1.63 ± 4.88 Propulsive mean force: − 0.06 ± 2.10 Anteroposterior net mean force: 0.08 ± 19.19 Vertical mean force: − 0.74 ± 1.82 Braking time: 0.85 ± 3.90 Propulsive time: − 0.60 ± 1.61 Functional asymmetry in: Countermovement jump height: − 0.67 ± 3.75 Session 2 Kinematic asymmetry in: Running velocity: 0.03 ± 0.04 Step length: − 0.22 ± 1.66 Step frequency: 0.24 ± 1.68 Stance time: − 0.09 ± 1.33 Flight time: − 0.45 ± 2.75 Kinetic asymmetry in: braking impulse: − 3.01 ± 4.12 Propulsive impulse: − 1.47 ± 2.99 Anteroposterior net impulse: 4.04 ± 13.28 Vertical impulse: − 1.12 ± 2.20 Braking mean force: − 3.49 ± 5.26 Propulsive mean force: − 1.55 ± 2.12 Anteroposterior net mean force: 3.94 ± 13.42 Vertical mean force: − 1.03 ± 1.78 Braking time: − 0.20 ± 3.37 Propulsive time: 0.08 ± 2.57 Functional asymmetry in: Countermovement jump height: − 0.76 ± 2.15	Changes in mean running velocity Vs. kinematic symmetry in: Running velocity: *r* = − 0.217, *p* = 0.477 Step length: *r* = − 0.096, *p* = 0.755 Step frequency: *r* = 0.094, *p* = 0.760 Stance time: *r* = 0.085, *p* = 0.783 Flight time: *r* = − 0.141, *p* = 0.645 Vs. kinetic symmetry in: Braking impulse: *r* = 0.249 *p* = 0.412 Propulsive impulse: *r* = 0.270, *p* = 0.372 Anteroposterior net impulse: *r* = 0.052, *p* = 0.867 Vertical impulse: *r* = 0.406, *p* = 0.169 Braking mean force: *r* = 0.055, *p* = 0.859 Propulsive mean force: *r* = 0.496, *p* = 0.085 Anteroposterior net mean force: *r* = 0.054, *p* = 0.860 Vertical mean force: *r* = 0.435, *p* = 0.138 Braking time: *r* = 0.249, *p* = 0.412 Propulsive time: *r* = − 0.130, *p* = 0.671 Vs. functional symmetry in: Countermovement jump height: *r* = 0.429, *p* = 0.137
Gołaś et al. [37] POL	*n* = 8 ♂: 0 ♀: 8 Age: ♂: NA ♀: 21.3 ± 5.4 Training status: elite level	EMG asymmery in: Quadriceps Hamstring Gluteus	50 m sprint time Lactate, 1 min post-set Lactate, 4 min post-set	$\% \mathrm{A}\mathrm{s}\mathrm{y}\mathrm{m}\mathrm{m}\mathrm{e}\mathrm{t}\mathrm{r}\mathrm{y}=$ $\frac{\left|\mathrm{R}\mathrm{M}\mathrm{S} \mathrm{l}\mathrm{e}\mathrm{f}\mathrm{t}-\mathrm{R}\mathrm{M}\mathrm{S} \mathrm{r}\mathrm{i}\mathrm{g}\mathrm{h}\mathrm{t}\right|}{\left(\mathrm{R}\mathrm{M}\mathrm{S} \mathrm{l}\mathrm{e}\mathrm{f}\mathrm{t}+\mathrm{R}\mathrm{M}\mathrm{S} \mathrm{r}\mathrm{i}\mathrm{g}\mathrm{h}\mathrm{t}\right)/2}\times 100$	Set 1: Quadriceps asymmetry: 10.7% Hamstring asymmetry: 12.6% Gluteus asymmetry: 15.9%	50 m sprint time Vs. quadriceps asymmetry: *r*_*s*_ = 0.58 Vs. hamstring asymmetry: *r*_*s*_ = 0.36 Vs. gluteus asymmetry: *r*_*s*_ = 0.59 Lactate 1 min post-set: Vs. quadriceps asymmetry: *r*_*s*_ = − 0.59, *p* = 0.0003 Vs. hamstring asymmetry: *r*_*s*_ = − 0.32 Vs. gluteus asymmetry: *r*_*s*_ = − 0.48, *p* = 0.0006 Lactate 4 min post-set: Vs. quadriceps asymmetry: *r*_*s*_ = − 0.46, *p* = 0.0074 Vs. hamstring asymmetry: *r*_*s*_ = − 0.19 Vs. gluteus asymmetry: *r*_*s*_ = − 0.43, *p* = 0.013
Haugen et al. [38] NOR	*n* = 22 ♂: NA ♀: NA Age: 23.0 ± 3.0 ♂: NA ♀: NA Training status: highly trained	Asymmetry in: Step velocity Step length Step rate Contact time Aerial time Touchdown angle Inter-thigh angle at TD TO angle Thigh angle at TO Knee angle at TO Maximal thigh flexion Range of thigh motion Knee flexion at maximal thigh extension Horizontal ankle velocity	Step velocity on a 18 m sprint with a 30–50 m self-selected in-run distance	$\% \mathrm{A}\mathrm{s}\mathrm{y}\mathrm{m}\mathrm{m}\mathrm{e}\mathrm{t}\mathrm{r}\mathrm{y}=$ $\frac{\mathrm{s}\mathrm{u}\mathrm{p}\mathrm{e}\mathrm{r}\mathrm{i}\mathrm{o}\mathrm{r} \mathrm{v}\mathrm{a}\mathrm{l}\mathrm{u}\mathrm{e}-\mathrm{i}\mathrm{n}\mathrm{f}\mathrm{e}\mathrm{r}\mathrm{i}\mathrm{o}\mathrm{r} \mathrm{v}\mathrm{a}\mathrm{l}\mathrm{u}\mathrm{e}}{\mathrm{i}\mathrm{n}\mathrm{f}\mathrm{e}\mathrm{r}\mathrm{i}\mathrm{o}\mathrm{r} \mathrm{v}\mathrm{a}\mathrm{l}\mathrm{u}\mathrm{e}}\times 100$	Step velocity: 0.6% Step length: 2.7% Step rate: 4.1% Contact time: 4.2% Aerial time: 4.9% Touchdown angle: NA Inter-thigh angle at TD: NA TO angle: NA Thigh angle at TO: NA Knee angle at TO: NA Maximal thigh flexion: NA Range of thigh motion: NA Knee flexion at maximal thigh extension: NA Horizontal ankle velocity: 7.3%	Step velocity on a 18 m sprint Vs. step velocity asymmetry: *r* = − 0.25, *p* > 0.05 Vs. step length asymmetry: *r* = − 0.37, *p* > 0.05 Vs. step frequency asymmetry: *r* = − 0.21, *p* > 0.05 Vs. contact time asymmetry: *r* = − 0.21, *p* > 0.05 Vs. flight time asymmetry: *r* = − 0.19, *p* > 0.05 Vs. touchdown angle asymmetry: *r* = 0.04, *p* > 0.05 vs. interthigh angle at TD asymmetry:: *r* = 0.09, *p* > 0.05 Vs. TO angle asymmetry: *r* = 0.03, *p* > 0.05 Vs. thigh angle at TO asymmetry: *r* = 0.27, *p* > 0.05 Vs. knee angle at TO asymmetry: *r* = 0.10, *p* > 0.05 Vs. maximal thigh flexion asymmetry: *r* = − 0.33, *p* > 0.05 Vs. range of thigh motion asymmetry: *r* = − 0.20, *p* > 0.05 Vs. knee flexion at maximal thigh extension asymmetry: *r* = − 0.40, *p* > 0.05 Vs. horizontal ankle velocity asymmetry: *r* = 0.06, *p* > 0.05
Nagahara et al. [6] JPN	*n* = 64 ♂: 32 ♀: 32 Age: ♂: 20.3 ± 2.0 ♀: 21.2 ± 3.9 Training status: Highly trained	Asymmetry in: Running velocity, Step length Step frequency Step width Stance time flight time Braking impulse Propulsive impulse Medial impulse Lateral impulse Mediolateral net impulse Anteroposterior net impulse Vertical impulse braking mean force Propulsive mean force Medial mean force Lateral mean force Mediolateral net mean force Anteroposterior net mean force Vertical mean force Braking time Propulsive time	Maximal sprinting velocity on a 60 m sprint	$\% \mathrm{A}\mathrm{s}\mathrm{y}\mathrm{m}\mathrm{m}\mathrm{e}\mathrm{t}\mathrm{r}\mathrm{y}=\frac{\frac{\mathrm{m}\mathrm{a}\mathrm{x}\mathrm{i}\mathrm{m}\mathrm{u}\mathrm{m} \mathrm{v}\mathrm{a}\mathrm{l}\mathrm{u}\mathrm{e}-\mathrm{m}\mathrm{i}\mathrm{n}\mathrm{i}\mathrm{m}\mathrm{u}\mathrm{m} \mathrm{v}\mathrm{a}\mathrm{l}\mathrm{u}\mathrm{e}}{\mathrm{m}\mathrm{a}\mathrm{x}\mathrm{i}\mathrm{m}\mathrm{u}\mathrm{m} \mathrm{v}\mathrm{a}\mathrm{l}\mathrm{u}\mathrm{e}}}{2}\times 100$	Running velocity: ♂: 0.2 ± 0.1% ♀: 0.2 ± 0.1% Step length: ♂: 4.6 ± 3.3% ♀: 3.1 ± 1.5% Step frequency: ♂: 4.7 ± 3.4% ♀: 3.1 ± 1.6% Step width: ♂: 31.1 ± 33.0% ♀: 36.7 ± 30.9% Stance time: ♂: 3.7 ± 2.3% ♀: 2.1 ± 1.6% Flight time: ♂: 6.1 ± 4.8% ♀: 4.9 ± 2.4% Braking impulse: ♂: 18.7 ± 15.0% ♀: 13.7 ± 11.4% Propulsive impulse: ♂: 7.7 ± 4.9% ♀: 7.0 ± 4.4% Medial impulse: ♂: 22.3 ± 14.5% ♀: 20.2 ± 16.1% Lateral impulse: ♂: 44.0 ± 41.2% ♀: 45.7 ± 38.2% Mediolateral net impulse: ♂: 9.2 ± 98.2% ♀: 0.9 ± 123.7% Anteroposterior net impulse: ♂: 39.8 ± 33.0% ♀: 46.0 ± 35.6% Vertical impulse: ♂: 8.4 ± 5.9% ♀: 5.3 ± 4.7% Braking mean force: ♂: 13.3 ± 10.3% ♀: 8.6 ± 7.8% Propulsive mean force: ♂: 8.2 ± 7.8% ♀: 8.3 ± 5.6% Medial mean force: ♂: 23.3 ± 17.1% ♀: 27.8 ± 20.9% Lateral mean force: ♂: 36.5 ± 30.8% ♀: 30.4 ± 24.1% Mediolateral net mean force: ♂: 10.5 ± 98.9% ♀: 0.7 ± 123.8% Anteroposterior net mean force: ♂: 41.3 ± 34.2% ♀: 46.7 ± 36.7% Vertical mean force: ♂: 6.0 ± 4.3% ♀: 4.3 ± 3.7% Braking time: ♂: 12.1 ± 14.4% ♀: 8.2 ± 7.1% Propulsive time: ♂: 4.7 ± 6.0% ♀: 4.3 ± 3.8%	Maximal sprinting velocity Vs. running velocity asymmetry: ♂: *r* = − 0.03, *p* = 0.88 ♀: *r* = 0.25, *p* = 0.17 Vs. step length asymmetry: ♂: *r* = − 0.25, *p* = 0.17 ♀: *r* = − 0.06, *p* = 0.74 Vs. step frequency asymmetry: ♂: *r* = − 0.24, *p* = 0.19 ♀: *r* = − 0.09, *p* = 0.62 Vs. step width asymmetry: ♂: *r* = 0.01, *p* = 0.94 ♀: *r* = 0.10, *p* = 0.58 Vs. stance time asymmetry: ♂: *r* = 0.18, *p* = 0.33 ♀: *r* = 0.03, *p* = 0.87 Vs. flight time asymmetry: ♂: *r* = − 0.31, *p* = 0.08 ♀: *r* = − 0.21, *p* = 0.25 Vs. braking impulse asymmetry: ♂: *r* = − 0.04, *p* = 0.82 ♀: *r* = 0.12, *p* = 0.53 Vs. propulsive impulse asymmetry: ♂: *r* = − 0.04, *p* = 0.84 ♀: *r* = 0.38, *p* = 0.16 Vs. medial impulse asymmetry: ♂: *r* = 0.10, *p* = 0.60 ♀: *r* = 0.15, *p* = 0.43 Vs. lateral impulse asymmetry: ♂: *r* = 0.16, *p* = 0.38 ♀: *r* = − 0.03, *p* = 0.86 Vs. mediolateral net impulse asymmetry: ♂: *r* = 0.04, *p* = 0.82 ♀: *r* = 0.09, *p* = 0.63 Vs. anteroposterior net impulse asymmetry: ♂: *r* = − 0.15, *p* = 0.42 ♀: *r* = 0.12, *p* = 0.51 Vs. vertical impulse asymmetry: ♂: *r* = 0.11, *p* = 0.53 ♀: *r* = 0.04, *p* = 0.85 Vs. braking mean force asymmetry: ♂: *r* = 0.05, *p* = 0.80 ♀: *r* = − 0.02, *p* = 0.91 Vs. propulsive mean force asymmetry: ♂: *r* = 0.17, *p* = 0.35 ♀: *r* = 0.24, *p* = 0.20 Vs. medial mean force asymmetry: ♂: *r* = − 0.02, *p* = 0.92 ♀: *r* = 0.09, *p* = 0.64 Vs. lateral mean force asymmetry: ♂: *r* = 0.10, *p* = 0.58 ♀: *r* = − 0.21, *p* = 0.24 Vs. mediolateral net mean force asymmetry: ♂: *r* = 0.05, *p* = 0.79 ♀: *r* = 0.09, *p* = 0.64 Vs. anteroposterior net mean force asymmetry: ♂: *r* = − 0.13, *p* = 0.47 ♀: *r* = 0.12, *p* = 0.52 Vs. vertical mean force asymmetry: ♂: *r* = 0.09, *p* = 0.63 ♀: *r* = 0.02, *p* = 0.90 Vs. braking time asymmetry: ♂: *r* = 0.19, *p* = 0.30 ♀: *r* = 0.27, *p* = 0.14 Vs. propulsive time asymmetry: ♂: *r* = 0.10, *p* = 0.59 ♀: *r* = 0.11, *p* = 0.29
Prvulović et al. [39] SRB	*n* = 18 ♂: NA ♀: 18 Age: 15.9 ± 1.3 Training status: highly trained	Explosive strength asymmetry during a countermovement jump	Mean velocity on a 100 m sprint test	$\mathrm{\%} \mathrm{A}\mathrm{s}\mathrm{y}\mathrm{m}\mathrm{m}\mathrm{e}\mathrm{t}\mathrm{r}\mathrm{y}= \frac{\mathrm{l}\mathrm{e}\mathrm{f}\mathrm{t} \mathrm{l}\mathrm{e}\mathrm{g} \mathrm{f}\mathrm{o}\mathrm{r}\mathrm{c}\mathrm{e}-\mathrm{r}\mathrm{i}\mathrm{g}\mathrm{h}\mathrm{t} \mathrm{l}\mathrm{e}\mathrm{g} \mathrm{f}\mathrm{o}\mathrm{r}\mathrm{c}\mathrm{e}}{\mathrm{s}\mathrm{u}\mathrm{m} \mathrm{o}\mathrm{f} \mathrm{f}\mathrm{o}\mathrm{r}\mathrm{c}\mathrm{e}}$	NA	Velocity on a 100 m sprint test Vs. explosive strength asymmetry: *p* > 0.05
Tottori et al. [43] JPN	*n* = 13 ♂: 10 ♀: 3 Age: 20.4 ± 1.7 Training status: highly trained	Asymmetry in cross-sectional area: • psoas major (L3 • L4, L4–L5, L5–S1, sum) • quadriceps femoris (30%, 50%, 70%, sum) • hamstrings (30%, 50%, 70%, sum)	Cross-directional difference in sprint time Averaged sprint time	$\mathrm{S}\mathrm{y}\mathrm{m}\mathrm{m}\mathrm{e}\mathrm{t}\mathrm{r}\mathrm{y} \mathrm{i}\mathrm{n}\mathrm{d}\mathrm{e}\mathrm{x} \left(\%\right)=2 \times \frac{\mathrm{r}\mathrm{i}\mathrm{g}\mathrm{h}\mathrm{t} \mathrm{s}\mathrm{i}\mathrm{d}\mathrm{e}-\mathrm{l}\mathrm{e}\mathrm{f}\mathrm{t} \mathrm{s}\mathrm{i}\mathrm{d}\mathrm{e}}{\mathrm{r}\mathrm{i}\mathrm{g}\mathrm{h}\mathrm{t} \mathrm{s}\mathrm{i}\mathrm{d}\mathrm{e}+\mathrm{l}\mathrm{e}\mathrm{f}\mathrm{t} \mathrm{s}\mathrm{i}\mathrm{d}\mathrm{e}} \times 100$	NA	Cross-directional difference in sprint time Vs. psoas major cross-sectional area L3–L4: *r* = − 0.541, *R*^2^ = 0.29, *p* = 0.056 L4–L5: *r* = − 0.550, *R*^2^ = 0.30, *p* = 0.052 L5–S1: *r* = − 0.495, *R*^2^ = 0.25, *p* = 0.084 SUM: *r* = − 0.614, *R*^2^ = 0.38, *p* = 0.026 Vs. quadriceps femoris cross-sectional area 30%: *r* = − 0.062, *R*^2^ = 0.00, *p* = 0.839 50%: *r* = − 0.399, *R*^2^ = 0.16, *p* = 0.177 70%: *r* = − 0.054, *R*^2^ = 0.00, *p* = 0.860 SUM: *r* = − 0.203, *R*^2^ = 0.04, *p* = 0.506 Vs. hamstrings cross-sectional area 30%: *r* = 0.292, *R*^2^ = 0.09, *p* = 0.333 50%: *r* = 0.190, *R*^2^ = 0.04, *p* = 0.535 70%: *r* = − 0.275, *R*^2^ = 0.08, *p* = 0.363 SUM: *r* = 0.138, *R*^2^ = 0.02, *p* = 0.654 Averaged sprint time Vs. psoas major symmetry:* r* = 0.092, *R*^2^ = 0.01, *p* = 0.765 vs. quadriceps femoris symmetry: *r* = 0.415, *R*^2^ = 0.17, *p* = 0.158 vs. hamstrings symmetry: *r* = 0.064, *R*^2^ = 0.00, *p* = 0.836
Trivers et al. [40] JM	Cohort 1996: *n* = 288 ♂: 155 ♀: 131 Cohort 2006: *n* = 163 ♂: 98 ♀: 65 Age: Cohort 2006: 22.6 ± 1.8 Training status: NA	Composite score asymmetry Asymmetry in: Knee joint width Ankle joint width Foot length	Mean velocity on a 90 m and 180 m sprint test	$\mathrm{F}\mathrm{l}\mathrm{u}\mathrm{c}\mathrm{t}\mathrm{u}\mathrm{a}\mathrm{t}\mathrm{i}\mathrm{n}\mathrm{g} \mathrm{a}\mathrm{s}\mathrm{y}\mathrm{m}\mathrm{m}\mathrm{e}\mathrm{t}\mathrm{r}\mathrm{y} = \frac{\left|\mathrm{v}\mathrm{a}\mathrm{l}\mathrm{u}\mathrm{e} \mathrm{l}\mathrm{e}\mathrm{f}\mathrm{t} \mathrm{l}\mathrm{e}\mathrm{g}-\mathrm{v}\mathrm{a}\mathrm{l}\mathrm{u}\mathrm{e} \mathrm{r}\mathrm{i}\mathrm{g}\mathrm{h}\mathrm{t} \mathrm{l}\mathrm{e}\mathrm{g}\right|}{\left(\mathrm{v}\mathrm{a}\mathrm{l}\mathrm{u}\mathrm{e} \mathrm{l}\mathrm{e}\mathrm{f}\mathrm{t} \mathrm{l}\mathrm{e}\mathrm{g}+\mathrm{v}\mathrm{a}\mathrm{l}\mathrm{u}\mathrm{e} \mathrm{r}\mathrm{i}\mathrm{g}\mathrm{h}\mathrm{t} \mathrm{l}\mathrm{e}\mathrm{g}\times 0.5\right)}$	Knee joint width: 1996: 0.007 ± 0.009% 2006: 0.016 ± 0.012% Ankle joint width: 1996: 0.008 ± 0.009 2006: 0.024 ± 0.021 Foot length: 1996: 0.008 ± 0.010% 2006: 0.020 ± 0.017%	Velocity on a 90 m sprint test Vs. composite score asymmetry: 1996: *R*^2^ = 0.02, *β* = 0.14, *p* = 0.09 2006: *R*^2^ = 0.03, *β* = 0.17, *p* = 0.05 Vs. knee joint width asymmetry: 1996: *R*^2^ = 0.04, *β* = 0.21, *p* = 0.01 2006: *R*^2^ = 0.04, *β* = 0.24, *p* = 0.01 Vs. ankle joint width asymmetry: 1996: *R*^2^ = NA, *β* = − 0.09, *p* = 0.26 2006: *R*^2^ = NA, *β* = 0.01, *p* = 0.90 Vs. foot length asymmetry: 1996: *R*^2^ = NA, *β* = − 0.11, *p* = 0.18 2006: *R*^2^ = NA, *β* = 0.07, *p* = 0.43 Velocity on a 180 m sprint test Vs. composite score asymmetry: 1996: *R*^2^ = 0.03, *β* = 0.18, *p* = 0.03 2006: *R*^2^ = 0.03, *β* = 0.17, *p* = 0.04 Vs. knee joint width asymmetry: 1996: *R*^2^ = 0.03, *β* = 0.19, *p* = 0.03 2006: *R*^2^ = 0.01, *β* = 0.14, *p* = 0.10 Vs. ankle joint width asymmetry: 1996: *R*^2^ = NA, *β* = − 0.00, *p* = 0.98 2006: *R*^2^ = NA, *β* = 0.07, *p* = 0.41 Vs. foot length asymmetry: 1996: *R*^2^ = NA, *β* = − 0.10, *p* = 0.23 2006: *R*^2^ = NA, *β* = 0.09, *p* = 0.30
Trivers et al. [41] JM	*n* = 73 ♂: NA ♀: NA Age: 23.0 ± 3.2 Training status: highly trained	Composite score asymmetry Asymmetry in: Knee joint width Ankle joint width Foot length	IAAF scores based on personal bests (100 m to 800 m), data collected over multiple seasons	$\mathrm{F}\mathrm{l}\mathrm{u}\mathrm{c}\mathrm{t}\mathrm{u}\mathrm{a}\mathrm{t}\mathrm{i}\mathrm{n}\mathrm{g} \mathrm{a}\mathrm{s}\mathrm{y}\mathrm{m}\mathrm{m}\mathrm{e}\mathrm{t}\mathrm{r}\mathrm{y} = \frac{\left|\mathrm{v}\mathrm{a}\mathrm{l}\mathrm{u}\mathrm{e} \mathrm{l}\mathrm{e}\mathrm{f}\mathrm{t} \mathrm{l}\mathrm{e}\mathrm{g}-\mathrm{v}\mathrm{a}\mathrm{l}\mathrm{u}\mathrm{e} \mathrm{r}\mathrm{i}\mathrm{g}\mathrm{h}\mathrm{t} \mathrm{l}\mathrm{e}\mathrm{g}\right|}{\left(\mathrm{v}\mathrm{a}\mathrm{l}\mathrm{u}\mathrm{e} \mathrm{l}\mathrm{e}\mathrm{f}\mathrm{t} \mathrm{l}\mathrm{e}\mathrm{g}+\mathrm{v}\mathrm{a}\mathrm{l}\mathrm{u}\mathrm{e} \mathrm{r}\mathrm{i}\mathrm{g}\mathrm{h}\mathrm{t} \mathrm{l}\mathrm{e}\mathrm{g}\times 0.5\right)}$	NA	IAAF scores Vs. composite score asymmetry: Partial *R*^2^ = 0.05, *β* = − 0.22, *p* = 0.03 Vs. knee width asymmetry: *R*^2^ = 0.05, *β* = − 0.22, *p* = 0.04 Vs. ankle width asymmetry: *R*^2^ = 0.03, *β* = − 0.17, *p* = 0.09 Vs. foot length asymmetry: *R*^2^ = 0.01, *β* = − 0.09, *p* = 0.28

*DCM TD* horizontal distance between the ground contact point at touchdown and the centre of mass, *DCM TO* horizontal distance between the ground contact point at toe-off and the centre of mass, *TD* touchdown, *TO* toe-off, *CM* centre of mass, *θSYM* symmetry angle, *NA* not available, *EMG* textile electromyography

## Discussion

This review synthesized evidence from 10 studies examining the relationship between functional, morphological, kinematic, and kinetic inter-limb asymmetries and sprinting performance in sprint athletes. Aside from a few findings indicating significant associations, most studies reported no significant relationships between inter‑limb asymmetry and sprinting performance metrics. Contrary to common assumptions, these findings suggest that greater asymmetry is not necessarily linked to poorer sprinting performance. A possible explanation is that athletes may adopt compensation strategies, whereby the dominant limb exerts greater force during propulsive phases to offset the weaker limb’s performance as was suggested from computer simulation studies [46, 47].

These results contrast with those of Fox et al.[18], whose meta-analysis identified significant but weak associations between inter-limb asymmetry and sprinting performance in mainly unilateral sports (*Zr* = 0.20, 95% CI 0.14–0.27; *r* = 0.20, *p* < 0.01), indicating that large asymmetries are associated with reduced sprinting performance. Methodological differences may account for these discrepancies. Specifically, Fox et al. [18] included predominantly team sport athletes engaged in unilateral sports such as soccer (eight of nine studies), a population likely to display larger asymmetries and, consequently, possibly greater susceptibility to performance decrements. Moreover, their review exclusively assessed functional asymmetry measures (e.g., SLCMJ, drop jump, and squat jump), whereas the present review also focused on morphological, kinematic, and kinetic asymmetries. Given the limited number of studies included and the metric-dependent nature of inter-limb asymmetry [48], the following discussion examines the results of individual studies across specific dimensions of inter-limb asymmetry.

### Functional Inter-limb Asymmetry and Sprinting Performance-Related Metrics

Gołaś et al. [34] investigated the relationship between lactate accumulation, sprinting performance, and inter-limb asymmetry, quantified using textile EMG embedded in specialized shorts, in eight elite female sprinters. Their findings showed that quadriceps and gluteal EMG asymmetries significantly decreased as lactate levels rose at 1 and 4 min post-exercise. The authors proposed that lactate may play a more active physiological role than being merely a metabolic by-product, which could contribute to more efficient muscle activation patterns and neuromuscular control [34]. Despite these valuable insights, the results should be interpreted with caution. The latter study did not assess the reliability of the EMG measurements, which is particularly important given that asymmetry is a ratio metric and therefore highly sensitive to measurement error in both limbs. Because inter-limb asymmetry was assessed using EMG sensors embedded in a garment, ensuring consistent placement over each specific muscle is challenging, which may further increase measurement error. Therefore, future researchers are encouraged to report study-specific reliability data, including coefficients of variation (CV), to enable differentiation between true inter-limb differences and measurement noise.

Prvulović et al. [36] observed no significant association between explosive strength asymmetry, assessed using a SLCMJ, and mean velocity in a 100 m sprint (*p* > 0.05). These results contrast with findings in soccer athletes, where greater SLCMJ asymmetry has been associated with impaired sprinting performance over 5 m, 10 m, and 20 m distances [14, 16, 49]. Two factors may explain the lack of association in the study by Prvulović et al. [36]. First, the sample size was relatively small, and no significant difference in asymmetry was observed between faster (1.32 ± 0.79%) and slower (2.87 ± 1.45%) sprinters, limiting the statistical power to detect a possible association. Second, asymmetry magnitudes were generally low compared to those reported in soccer cohorts. For example, Bishop et al. [49] documented SLCMJ asymmetries of up to 18%, suggesting that only larger asymmetry magnitudes, possibly linked to the unilateral demands of soccer, are sufficient to be negatively associated with sprinting performance. Despite these advances in understanding, it is important to recognize that cross-sectional study designs limit the ability to infer causality. To address this limitation, Gleadhill et al. [33] conducted a longitudinal assessment in which sprinting performance over 60 m and inter-limb jump-height asymmetry, measured via a SLCMJ, were tested one month apart. Their findings showed that small fluctuations in inter-limb asymmetry were not significantly associated with changes in sprinting performance. This suggests that short-term variations in asymmetry likely reflect normal neuromuscular variability rather than meaningful mechanical inefficiency, and therefore may not be inherently detrimental to sprinting performance.

### Morphological Inter-limb Asymmetry and Sprinting Performance-Related Metrics

Sprinting performance has been reported to be positively associated with psoas major and cross-sectional area [50]. Sprint athletes also typically exhibit greater quadriceps femoris and hamstrings cross-sectional areas compared to non-sprinters [51, 52]. However, Tottori et al. [37] found that inter-limb asymmetry in quadriceps and hamstrings cross-sectional area was not significantly associated with curve-sprinting performance. In their study, athletes performed curve sprints in both counter-clockwise and clockwise directions, allowing the researchers to calculate the difference in sprinting time across directions. Their results indicated that inter-limb asymmetry in psoas major cross-sectional area was negatively associated with these cross-directional differences. Specifically, athletes with a larger psoas major on the right limb (i.e. the outer leg during counter-clockwise running) ran faster in the counter-clockwise direction than in the clockwise direction. These findings highlight that the potential influence of inter-limb asymmetry is highly task-dependent. Moreover, the direction of the asymmetry itself, such as having a larger muscle on the inside versus outside limb during a curve, may determine whether the asymmetry is potentially beneficial or detrimental. For instance, previous research showed that during curve sprinting, the inside leg consistently produces lower peak forces than the outside leg, naturally generating larger asymmetries [53]. Thus, asymmetry in certain muscles may therefore play a meaningful role in curvilinear running but not necessarily in straight-line sprinting, suggesting that both movement types and the directional nature of the asymmetry should be assessed independently when evaluating the functional consequences of inter-limb differences.

Trivers et al. [38, 39] investigated knee and ankle joint width asymmetry as well as foot length asymmetry in relation to sprinting performance. Jamaican sprinters exhibited greater knee joint width asymmetry than non-runners (*p* < 0.001), and this asymmetry was significantly associated with poorer performance (i.e. lower mean velocities over 90 m and 180 m, and lower IAAF scores). By contrast, ankle width and foot length asymmetry were not significantly related to sprinting performance. The authors speculated that, alongside factors such as the proportion and quality of fast-twitch muscle fibres, symmetrical and stable lower limbs may play a role in enhancing sprinting success [38, 39]. The studies by Trivers et al. [38, 39] also employed composite scores for determining overall asymmetry and found a significant negative relationship with sprinting performance. Although these findings may appear to suggest that lower overall asymmetry aligns with better sprinting performance, it is generally not recommended to calculate composite scores. This is because inter-limb asymmetry varies across different body regions, tools and metrics used for assessment [23, 48, 54, 55]. Moreover, composite values can obscure interpretation by conflating large asymmetries in a few variables with small asymmetries across many variables [56]. As a result, assessing each type of asymmetry independently is considered a more valid and informative approach than aggregating them into a single overall score.

### Kinematic Inter-limb Asymmetry and Sprinting Performance-Related Metrics

Kinematic asymmetry appears to be sex specific. For example, Bissas et al. [13] observed a strong negative association between 100 m sprinting time and foot vertical velocity pre-touchdown asymmetry in world-class male sprinters (*r* = − 0.86), whereas in world-class female sprinters, shank angle touchdown asymmetry was positively associated with 100 m sprinting time (*r* = 0.81). Similarly, Nagahara et al. [6] found that flight-time asymmetry was significantly associated with maximal sprinting velocity during a 60-m sprint in males, while no significant association was observed in female sprinters.

Previous analyses of Usain Bolt’s sprinting mechanics have demonstrated pronounced inter-limb asymmetries. For example, his right leg has been shown to produce approximately 13% greater peak force, while his left leg exhibits a ~ 14% longer ground-contact time [57]. Another investigation similarly reported notable step-length discrepancies between limbs in Bolt’s kinematics [58]. These observations illustrate that even the world’s fastest sprinter does not display perfect symmetry. In line with this, the findings of the present systematic review indicate that kinematic inter-limb asymmetry seems not to be associated with determinants of sprinting performance or actual measures of sprinting performance [6, 13, 32, 35]. In addition to these cross-sectional observations, Gleadhill et al. [33] found that changes in mean velocity during a 60-m sprint were not significantly associated with changes in kinematic asymmetry over time. Beyond potential compensatory strategies that may mitigate the effects of asymmetry, it is also possible that group-level analyses lack the sensitivity to detect meaningful relationships. In other words, some athletes may display lower inter-limb asymmetry alongside superior sprinting performance, whereas in others, asymmetry may show no clear relationship or could even be associated with enhanced performance [32]. Further supporting this view, Exell et al. [32] found that composite scores of overall kinematic asymmetry were not significantly associated with mean sprinting velocity during a 60 m sprint. Similar to the findings on morphological inter-limb asymmetry, these findings suggest that while isolated asymmetries in specific variables may be associated with performance under certain conditions, global measures of asymmetry may lack sensitivity in predicting sprinting ability.

### Kinetic Inter-limb Asymmetry and Sprinting Performance-Related Metrics

Exell et al. [32] reported a negative association between relative vertical stiffness asymmetry and sprinting performance (*r*_*s*_ = − 0.24), alongside positive associations between net ankle work asymmetry (*r* = 0.90) and maximum vertical asymmetry (*r* = 0.76) with mean velocity over a 60 m sprint. However, the authors emphasized that these relationships were highly athlete-specific. For example, two athletes with comparable inter-limb kinetic asymmetries demonstrated markedly different step velocities, suggesting that sensitivity to asymmetry may vary between individuals [32]. This variation may stem from the ability of certain athletes to effectively compensate for asymmetries, whereas others may lack such compensatory mechanisms. Consistent with findings on morphological and kinematic measures, kinetic composite scores were not significantly associated with sprinting performance [32]. Similarly, Nagahara et al. [6] found no significant relationships between the magnitude of inter-limb kinetic asymmetry across all assessed variables and maximal sprinting speed over 60 m. Likewise, Gleadhill et al. [33] showed that changes in running speed were not significantly associated with changes in kinetic asymmetry over two test occasions. Collectively, these results suggest that biomechanical asymmetries may not be a major determinant of sprinting performance and that their impact is highly individual, irrespective of performance level. Consequently, athletes may not need to prioritize correcting these asymmetries within their training routine in view of performance enhancement. However, if practitioners consider asymmetry reduction desirable for other reasons, such as injury prevention, existing evidence indicates that targeted strengthening of the weaker limb can effectively increase its capacity and reduce inter‑limb asymmetry [59].

### Limitations and Directions for Future Research

Substantial variability existed across studies in the dimensions of asymmetry assessed (functional, morphological, kinematic, and kinetic), the metrics and equations used to calculate asymmetry, and participant characteristics (sex, age, and training status). The small number of eligible studies combined with the high between-study heterogeneity made a meta-analysis inappropriate. Although most studies scored moderate-to-high on the quality assessment (6–8/10), all findings scored very low on certainty of evidence. Moreover, only 3 of the 10 assessed the reliability of their asymmetry measures, making it difficult to determine whether reported values reflect true side-to-side differences or are partly attributable to measurement error [23]. With the exception of one study [33], all included research employed cross-sectional designs, precluding any causal inference. Future research should consistently report reliability data (e.g., intraclass correlation coefficients and correlation of variance values), establish task-, metric-, and muscle-specific thresholds for performance decrements while accounting for individual responses (i.e. responders vs. non-responders), and avoid the use of composite scores. Finally, standardization in asymmetry calculation methods is warranted, as outlined by Bishop et al. [60].

## Conclusion

The limited number of studies and high heterogeneity in asymmetry scores and sample sizes made it difficult to draw definitive conclusions. Overall, the available evidence suggests that inter-limb asymmetry is generally not significantly associated with sprinting performance in sprint athletes. However, specific asymmetries in certain metrics may show negative associations with performance under particular conditions. Future research should employ task-, metric-, and muscle-specific assessments and use robust longitudinal designs to clarify the practical relevance of inter-limb differences for sprinting performance.

## Supplementary Information

Supplementary Material 1.

## Data Availability

Not applicable.

## Abbreviations

SLCMJ: Single-leg countermovement jump
PRISMA: Preferred Reporting Items for Systematic Reviews and Meta-Analyses

## References

[1] Savriama Y, Klingenberg CP. Beyond bilateral symmetry: geometric morphometric methods for any type of symmetry. BMC Evol Biol. 2011. 10.1186/1471-2148-11-280. PMC3209460 21958045

[2] Maloney SJ. The relationship between asymmetry and athletic performance: a critical review. J Strength Cond Res. 2019;33(9):2579–93. 10.1519/JSC.0000000000002608 29742749

[3] D’Hondt J, Chapelle L, Van Droogenbroeck L, Aerenhouts D, Clarys P, D’Hondt E. Bioelectrical impedance analysis as a means of quantifying upper and lower limb asymmetry in youth elite tennis players: an explorative study. Eur J Sport Sci. 2022;22(9):1343–54. 10.1080/17461391.2021.1960624 34296664

[4] Bishop C, de Keijzer KL, Turner AN, Beato M. Measuring interlimb asymmetry for strength and power: a brief review of assessment methods, data analysis, current evidence, and practical recommendations. J Strength Cond Res. 2023;37(3):745–50. 10.1519/JSC.0000000000004384 36695841

[5] D’Hondt J, Chapelle L. Change of direction asymmetry in youth elite tennis players: a longitudinal study. Int J Sports Med. 2024;45(6):436–42. 10.1055/a-2231-9630 38109926

[6] Nagahara R, Gleadhill S. Asymmetries of kinematics and kinetics in female and male sprinting. J Sport Med Phys Fit. 2023;63(8):891–8. 10.23736/s0022-4707.23.14778-5 37166253

[7] Pitsiladis Y, Davis A, Johnson D. The science of speed: determinants of performance in the 100 m sprint a commentary. Int J Sports Sci Coach. 2011;6(3):495–8. 10.1260/1747-9541.6.3.49

[8] Smirniotou A, Katsikas C, Paradisis G, Argeitaki P, Zacharogiannis E, Tziortzis S. Strength-power parameters as predictors of sprinting performance. J Sports Med Phys Fitness. 2008;48(4):447–54. 18997647

[9] Hakkinen K, Keskinen KL. Muscle cross-sectional area and voluntary force production characteristics in elite strength- and endurance-trained athletes and sprinters. Eur J Appl Physiol Occup Physiol. 1989;59(3):215–20. 10.1007/BF02386190 2583165

[10] Cormie P, McGuigan MR, Newton RU. Developing maximal neuromuscular power: part 2—training considerations for improving maximal power production. Sports Med. 2011;41(2):125–46. 10.2165/11538500-000000000-00000 21244105

[11] Bobbert MF, de Graaf WW, Jonk JN, Casius LJR. Explanation of the bilateral deficit in human vertical squat jumping. J Appl Physiol. 2006;100(2):493–9. 10.1152/japplphysiol.00637.2005 16239616

[12] Taniguchi Y. Relationship between the modifications of bilateral deficit in upper and lower limbs by resistance training in humans. Eur J Appl Physiol Occup Physiol. 1998;78(3):226–30. 10.1007/s004210050411 9721000

[13] Bissas A, Walker J, Paradisis GP, Hanley B, Tucker CB, Jongerius N, et al. Asymmetry in sprinting: an insight into sub-10 and sub-11 s men and women sprinters. Scand J Med Sci Spor. 2022;32(1):69–82. 10.1111/sms.14068 34587293

[14] Bishop C, Brashill C, Abbott W, Read P, Lake J, Turner A. Jumping asymmetries are associated with speed, change of direction speed, and jump performance in elite academy soccer players. J Strength Cond Res. 2021;35(7):1841–7. 10.1519/JSC.0000000000003058 30707141

[15] Bishop C, Read P, Brazier J, Jarvis P, Chavda S, Bromley T, et al. Effects of interlimb asymmetries on acceleration and change of direction speed: a between-sport comparison of professional soccer and cricket athletes. J Strength Cond Res. 2021;35(8):2095–101. 10.1519/JSC.0000000000003135 31008864

[16] Fort-Vanmeerhaeghe A, Bishop C, Buscà B, Aguilera-Castells J, Vicens-Bordas J, Gonzalo-Skok O. Inter-limb asymmetries are associated with decrements in physical performance in youth elite team sports athletes. PLoS ONE. 2020;15(3):e0229440. 10.1371/journal.pone.0229440 PMC7053777 32126107

[17] D'Hondt J, Denaryer R, Verstraeten S, Clarys P, Chapelle L. In-Water 505 Deficit as an Isolated Measure of Directional Performance and Asymmetry in Male Water Polo Athletes. Int J Sports Physiol. Perform. 2026;1–7. https://journals.humankinetics.com/view/journals/ijspp/aop/article-10.1123-ijspp.2025-0677/article-10.1123-ijspp.2025-0677.xml 10.1123/ijspp.2025-0677 42501976

[18] Fox KT, Pearson LT, Hicks KM. The effect of lower inter-limb asymmetries on athletic performance: a systematic review and meta-analysis. PLoS ONE. 2023;18(6):e0286942. 10.1371/journal.pone.0286942 PMC10249853 37289826

[19] Chapelle L, Bishop C, D’Hondt J, D’Hondt E, Clarys P. Morphological and functional asymmetry in elite youth tennis players compared to sex- and age-matched controls. J Sports Sci. 2022;40(14):1618–28. 10.1080/02640414.2022.2096769 35830516

[20] Carpes FP, Mota CB, Faria IE. On the bilateral asymmetry during running and cycling—a review considering leg preference. Phys Ther Sport. 2010;11(4):136–42. 10.1016/j.ptsp.2010.06.005 21055708

[21] Gonzalez MP, Montalvo S, Dietze-Hermosa M, Gomez M, Dorgo S. A comparison of morphological, jump, and sprint kinematic asymmetries in division I track and field athletes. Int J Exerc Sci. 2023;16(1):1306–19. 10.70252/TFBN4736 PMC10824295 38288257

[22] D'Hondt J, Chapelle L, Bishop C, Aerenhouts D, De Pauw K, Clarys P, et al. Test-retest and inter-rater reliability of a field-based test battery to assess lower limb isometric strength and inter-limb asymmetry in healthy adult distance runners of various training levels. Research Square. 10.21203/rs.3.rs-9354122/v1

[23] D’Hondt J, Chapelle L, Bishop C, Aerenhouts D, De Pauw K, Clarys P, et al. Association between inter-limb asymmetry and determinants of middle- and long-distance running performance in healthy populations: a systematic review. Sports Med-Open. 2024;10(1):127. 10.1186/s40798-024-00790-w PMC11599690 39589611

[24] D'Hondt J, Denaryer R, Verstraeten S, Clarys P, Chapelle L. Variations in lower‑limb body composition asymmetry and leg length discrepancy by sex and training level, and their association with performance in recreational endurance runners: a cross‑sectional study. BMC Sports Sci. Med. Rehab. 2026.

[25] Page MJ, Moher D, Bossuyt PM, Boutron I, Hoffmann TC, Mulrow CD, et al. PRISMA 2020 explanation and elaboration: updated guidance and exemplars for reporting systematic reviews. BMJ. 2021;372:n160. 10.1136/bmj.n160 PMC8005925 33781993

[26] Ouzzani M, Hammady H, Fedorowicz Z, Elmagarmid A. Rayyan-a web and mobile app for systematic reviews. Syst Rev. 2016;5(1):210. 10.1186/s13643-016-0384-4 PMC5139140 27919275

[27] McKay AKA, Stellingwerff T, Smith ES, Martin DT, Mujika I, Goosey-Tolfrey VL, et al. Defining training and performance caliber: a participant classification framework. Int J Sport Physiol. 2022;17(2):317–31. 10.1123/ijspp.2021-0451 34965513

[28] Downs SH, Black N. The feasibility of creating a checklist for the assessment of the methodological quality both of randomised and non-randomised studies of health care interventions. J Epidemiol Commun H. 1998;52(6):377–84. 10.1136/jech.52.6.377 PMC1756728 9764259

[29] Jarvis P, Turner A, Read P, Bishop C. Reactive strength index and its associations with measures of physical and sports performance: a systematic review with meta-analysis. Sports Med. 2022;52(2):301–30. 10.1007/s40279-021-01566-y 34606061

[30] Sarmento H, Clemente FM, Araújo D, Davids K, McRobert A, Figueiredo A. What performance analysts need to know about research trends in association football (2012–2016): a systematic review. Sports Med. 2018;48(4):799–836. 10.1007/s40279-017-0836-6 29243038

[31] Zeng LN, Brignardello-Petersen R, Hultcrantz M, Siemieniuk RAC, Santesso N, Traversy G, et al. GRADE guidelines 32: GRADE offers guidance on choosing targets of GRADE certainty of evidence ratings. J Clin Epidemiol. 2021;137:163–75. 10.1016/j.jclinepi.2021.03.026 33857619

[32] Exell T, Irwin G, Gittoes M, Kerwin D. Strength and performance asymmetry during maximal velocity sprint running. Scand J Med Sci Sports. 2017;27(11):1273–82. 10.1111/sms.12759 27671707

[33] Gleadhill S, Nagahara R. Asymmetry changes in sprinting do not relate to changes in sprint performance. Int J Sports Sci Coach. 2025. 10.1177/17479541251377991.

[34] Golas A, Terbalyan A, Gepfert M, Roczniok R, Matusinski A, Kotula K, et al. repeated sprint performance and inter-limb asymmetry in elite female sprinters: a study of lactate dynamics and lower limb muscle activity. J Funct Morphol Kinesiol. 2025;10(2):213. 10.3390/jfmk10020213 PMC12194348 40566463

[35] Haugen T, Danielsen J, McGhie D, Sandbakk O, Ettema G. Kinematic stride cycle asymmetry is not associated with sprint performance and injury prevalence in athletic sprinters. Scand J Med Sci Sports. 2018;28(3):1001–8. 10.1111/sms.12953 28759127

[36] Prvulovic N, Coh M, Cular D, Tomljanovic M, Sporis G, Fiser SZ. countermovement jump in female sprinters: kinetic parameters and asymmetry. Symmetry-Basel. 2022;14(6):1130. 10.3390/sym14061130

[37] Tottori N, Kurihara T, Otsuka M, Isaka T. Relationship between lateral differences in the cross-sectional area of the psoas muscle and curve running time. J Physiol Anthropol. 2016;35:3. 10.1186/s40101-016-0086-6 PMC4727261 26810310

[38] Trivers R, Palestis BG, Manning JT. The symmetry of children’s knees is linked to their adult sprinting speed and their willingness to sprint in a long-term Jamaican study. PLoS ONE. 2013;8(8):e72244. 10.1371/journal.pone.0072244. PMC3747165 23977263

[39] Trivers R, Fink B, Russell M, McCarty K, James B, Palestis BG. Lower body symmetry and running performance in elite Jamaican track and field athletes. PLoS ONE. 2014. 10.1371/journal.pone.0113106. PMC4234648 25401732

[40] Egger M, Smith GD, Schneider M, Minder C. Bias in meta-analysis detected by a simple, graphical test. Bmj-Brit Med J. 1997;315(7109):629–34. 10.1136/bmj.315.7109.629 PMC2127453 9310563

[41] Guyatt G, Oxman AD, Akl EA, Kunz R, Vist G, Brozek J, et al. GRADE guidelines: 1. Introduction-GRADE evidence profiles and summary of findings tables. J Clin Epidemiol. 2011;64(4):383–94. 10.1016/j.jclinepi.2010.04.026 21195583

[42] Zhang Y, Coello PA, Guyatt GH, Yepes-Nunez JJ, Akl EA, Hazlewood G, et al. GRADE guidelines: 20. Assessing the certainty of evidence in the importance of outcomes or values and preferences-inconsistency, imprecision, and other domains. J Clin Epidemiol. 2019;111:83–93. 10.1016/j.jclinepi.2018.05.011 29800687

[43] Guyatt GH, Oxman AD, Kunz R, Brozek J, Alonso-Coello P, Ring D, et al. GRADE guidelines 6. Rating the quality of evidence-imprecision. J Clin Epidemiol. 2011;64(12):1283–93. 10.1016/j.jclinepi.2011.01.012 21839614

[44] Zhang Y, Alonso-Coello P, Guyatt GH, Yepes-Nuñez JJ, Akl EA, Hazlewood G, et al. GRADE guidelines: 19. Assessing the certainty of evidence in the importance of outcomes or values and preferences-risk of bias and indirectness. J Clin Epidemiol. 2019;111:94–104. 10.1016/j.jclinepi.2018.01.013 29452223

[45] Clemente FM, Scanlan AT, Ramirez-Campillo R, Martinho D, Thapa RK, Chamari K, et al. The correlations between training load parameters and physical performance adaptations in team sports: a systematic review and meta-analysis. Sports Med-Open. 2025;11(1):156. 10.1186/s40798-025-00952-4 PMC12698923 41381923

[46] Yoshioka S, Nagano A, Hay DC, Fukashiro S. The effect of bilateral asymmetry of muscle strength on jumping height of the countermovement jump: a computer simulation study. J Sports Sci. 2010;28(2):209–18. 10.1080/02640410903428566 20054738

[47] Yoshioka S, Nagano A, Hay DC, Fukashiro S. The effect of bilateral asymmetry of muscle strength on the height of a squat jump: a computer simulation study. J Sports Sci. 2011;29(8):867–77. 10.1080/02640414.2011.568512 21506038

[48] D'Hondt J, Vanhoudt M, Maluta D, Chapelle L, Aerenhouts D. Does a muscle fatigue-inducing protocol alters the magnitude of jump inter-limb asymmetry in healthy adolescents? J Hum Kinet. 2026;103:57–68. 10.5114/jhk/205705 PMC13551332 42712858

[49] Bishop C, Turner A, Maloney S, Lake J, Loturco I, Bromley T, et al. Drop jump asymmetry is associated with reduced sprint and change-of-direction speed performance in adult female soccer players. Sports (Basel). 2019. 10.3390/sports7010029. PMC6359266 30669686

[50] Copaver K, Hertogh C, Hue O. The effects of psoas major and lumbar lordosis on hip flexion and sprint performance. Res Q Exercise Sport. 2012;83(2):160–7. 10.1080/02701367.2012.10599846 22808701

[51] Hoshikawa Y, Muramatsu M, Iida T, Uchiyama A, Nakajima Y, Kanehisa H. Event-related differences in the cross-sectional areas and torque generation capabilities of quadriceps femoris and hamstrings in male high school athletes. J Physiol Anthropol. 2010;29(1):13–21. 10.2114/jpa2.29.13 20453429

[52] Hoshikawa Y, Muramatsu M, Iida T, Ii N, Nakajima Y, Kanehisa H. Sex differences in the cross-sectional areas of psoas major and thigh muscles in high school track and field athletes and nonathletes. J Physiol Anthropol. 2011;30(2):47–53. 10.2114/jpa2.30.47 21483176

[53] Chang YH, Kram R. Limitations to maximum running speed on flat curves. J Exp Biol. 2007;210(6):971–82. 10.1242/jeb.02728 17337710

[54] D'Hondt J, Chapelle L, Lindekens T, Clarys P. Whole-body morphological inter-limb asymmetry in adult judokas according to competition level, weight category and sex. BMC Sports Sci Med Rehabil. 2025;17:380. 10.1186/s13102-025-01440-8 PMC12751752 41286933

[55] D'Hondt J, Chapelle L, Pletinckx B, Sadouni M, Bosman L, Lindekens T, Clarys P. Intra- and inter-limb isokinetic strength asymmetry in competitive judokas: differences in magnitude across competition levels and sex. J Human Kinetic. Accepted.

[56] Exell TA, Gittoes MJR, Irwin G, Kerwin DG. Gait asymmetry: composite scores for mechanical analyses of sprint running. J Biomech. 2012;45(6):1108–11. 10.1016/j.jbiomech.2012.01.007 22296935

[57] Something strange in Usain Bold's stride 2017. https://www.nytimes.com/2017/07/20/sports/olympics/usain-bolt-stride-speed.html

[58] Stuhec S, Planjsek P, Coh M, Mackala K. Multicomponent velocity measurement for linear sprinting: usain bolt’s 100 m world-record analysis. Bioeng Basel. 2023;10(11):1254. 10.3390/bioengineering10111254 PMC10669785 38002378

[59] Brown SR, Feldman ER, Cross MR, Helms ER, Marrier B, Samozino P, et al. The potential for a targeted strength-training program to decrease asymmetry and increase performance: a proof of concept in sprinting. Int J Sport Physiol. 2017;12(10):1392–5. 10.1123/ijspp.2016-0590 28338355

[60] Bishop C, Read P, Chavda S, Turner A. Asymmetries of the lower limb: the calculation conundrum in strength training and conditioning. Strength Cond J. 2016;38(6):27–32. 10.1519/SSC.0000000000000264

