# Supplementary material for: The Relationship Between Inter-limb Asymmetry and Sprinting Performance in Sprint Athletes: A Systematic Review
Source: Sports Med Open. 2026 Oct 1;12:146. doi: 10.1186/s40798-026-01082-1 (PMC13631215; doi:10.1186/s40798-026-01082-1)
Supplement: Supplementary file 1 [file 40798_2026_1082_MOESM1_ESM.docx]

Table S1. Summary of Studies Excluded at Full‑Text Review

| **Author** | **Reason for exclusion** |
| --- | --- |
| Girard [1] | Not an original research article. |
| Inal et al. [2] | No association reported between inter‑limb asymmetry and sprint performance. |
| Lockie et al. [3] | Wrong study population: strength trained men instead of sprint athletes |
| Mackala et al. [4] | No association reported between inter‑limb asymmetry and sprint performance. |
| Vellucci et al. [5] | Wrong study population: included athletes from team sports (rugby, soccer, ice hockey). |
| Vagenas et al. [6] | No association reported between inter‑limb asymmetry and sprint performance. |

**References:**

1.	Girard O. Asymmetry in sprinting: The myth of perfection and the reality of performance. J Sport Health Sci. 2025 Dec;14.

2.	Inal HS, Erbug B, Kotzamanidis C. Sprinting, isokinetic strength, and range of motion of ankle joints in Turkish male and female national sprinters may have a relationship. Turk J Med Sci. 2012 Dec;42(6):1098-104.

3.	Lockie RG, Risso FG, Lazar A, Giuliano DV, Stage AA, Liu TM, et al. Between-Leg Mechanical Differences as Measured by the Bulgarian Split-Squat: Exploring Asymmetries and Relationships with Sprint Acceleration. Sports (Basel). 2017 Sep 1;5(3).

4.	Mackala K, Michalski R, Coh M. Asymmetry of Step Length in Relationship to Leg Strength in 200 meters Sprint of different Performance Levels. Journal of Human Kinetics. 2010 Sep;25:101-8.

5.	Vellucci CL, Beaudette SM. A need for speed: Objectively identifying full-body kinematic and neuromuscular features associated with faster sprint velocities. Front Sports Act Liv. 2023 Feb 3;4.

6.	Vagenas G, Hoshizaki TB. Optimization of an Asymmetrical Motor Skill: Sprint Start. J Appl Biomech. 1968;2(1):29-40.
